# 2-hydroxylated sphingomyelin profiles in cells from patients with mutated fatty acid 2-hydroxylase

**DOI:** 10.1186/1476-511X-10-84

**Published:** 2011-05-20

**Authors:** Phyllis Dan, Simon Edvardson, Jacek Bielawski, Hiroko Hama, Ann Saada

**Affiliations:** 1 Department of Genetics and Metabolic Diseases and the Monique and Jacques Roboh Department of Genetic Research, Hadassah-Hebrew University Medical Center, Jerusalem, Israel; 2 Pediatric Neurology Unit Hadassah-Hebrew University Medical Center, Jerusalem, Israel; 3 Department of Biochemistry and Molecular Biology, Medical University of South Carolina, Charleston SC, USA

**Keywords:** Fatty acid hydroxylase, hydroxylated fatty acid sphingomyelin, autosomal recessive leukodystrophy

## Abstract

Fatty acid 2-hydroxylase (FA2H) is the enzyme responsible for the hydroxylation of free fatty acids prior to their incorporation into 2-hydroxylated sphingolipids, which are the major constituents of the myelin leaflet. Mutated FA2H has been associated with neurodegenerative diseases. Decreased FA2H activity was demonstrated only *in vitro*, but not in patient tissues. In this study we characterized the 2-hydroxylated sphingomyelin (SM) profiles in blood and fibroblasts from patients harboring a deleterious FA2H mutatation, and found that hydroxylated fatty acid sphingomyelin is present in normal amounts in patient lymphocytes, but decreased to a different extent in fibroblasts and erythrocytes.

## Background

A compact myelin sheet surrounding the axon is essential for correct nerve conduction [1]. Myelin is composed of over 70% lipids, the most abundant of which are the galactolipids galactosylceramide (GalC) and its sulfated form, sulfatide. As more than fifty percent of GalC and sulfatide are hydroxylated at the C2 position on the fatty acid (FA) moiety, it has been estimated that approximately twenty five percent of the outer leaflet lipids in myelin are hydroxylated [2,3]. Based on studies of model membranes, hydroxylation likely contributes to the stability of myelin by virtue of the hydrogen bonding between the hydroxy groups, the galactose head group and the polar part of the ceramide backbone [4].

The 2- hydroxylation of sphingolipids [for review, see [5]] occurs during de novo ceramide synthesis and is catalyzed by the enzyme fatty acid 2-hydroxylase (FA2H), a membrane-bound protein containing a cytochrome b5-like heme-binding domain responsible for the redox activity, and a sterol desaturase domain [6]. The products of FA2H are free 2-hydroxy fatty acids (OHFA), which are subsequently incorporated into ceramide, the precursor of galactosylceramide [7]. FA2H-deficient mice lacking hydroxylated fatty acids in the central and peripheral nervous system had normal neuronal development, but showed late onset axon and myelin sheet degeneration [8] and exhibited CNS dysfunction [9].

Mutations in the human *FA2H *gene were identified in patients with autosomal recessive leukodystrophy characterized by childhood (4-5 y) onset spasticity, dystonia and white matter degeneration. In seven patients, the desaturase domain was disrupted by a splice site mutation causing the skipping of exons 5 and 6 while a missense mutation in a conserved residue was detected in the other two patients [10]. Recently, mutated FA2H was also found to be the underlying cause of a complicated hereditary spastic paraplegia (SPG35) [11], and neurodegeneration with brain iron accumulation [12]. Although in vitro transfection studies of mutated FA2H disclosed reduced hydroxy fatty acid synthesis, decreased enzymatic activity was not demonstrated in patients. In fact, tetracosanoic acid hydroxylating activity in patient fibroblasts was indistinguishable from that of normal controls [10]. Moreover, the impact of defective FA2H on fatty acid composition in patients is (to our knowledge) unknown. The aim of this study was to characterize the functional impact of FA2H splice site mutation on the fatty acid and hydroxy fatty acid sphingomyelin profiles in patient's blood and fibroblasts.

## Methods

Cell culture reagents were obtained from Biological Industries, Beit HaEmek, Israel. Hisopaque and all other reagents were from Sigma-Aldrich, Israel.

### Subjects

Blood from two patients and fibroblasts from one patient harboring the FA2H c.786+1G→A mutation (family 1, described by Edvardsson et al [10] and from three controls were obtained with informed consent and approval from the local IRB.

### Lymhocyte isolation

Lymphocytes were isolated from whole blood (EDTA anti-coagulant) using Histopaque-1077 according to the manufacturer's instructions. The resulting pellet was lyophilized prior to lipid analysis.

### Erythrocyte membrane preparation

Erythrocyte membranes were prepared from whole blood collected with heparin as anti-coagulant. The blood was spun at 1000 × g for 10 min, the plasma was removed and the erythrocytes were washed three times with twice their volume of saline. Subsequently, cells were hemolyzed in 5 ml of water then spun at 12000 × g for 10 minutes. The membranes were washed twice with water and lyophilized prior to lipid analysis.

### Cell culture

Primary fibroblasts were grown in DMEM (high glucose) supplemented with 15% fetal calf serum in the presence of penicillin and streptomycin. Confluent cells were harvested by trypsinization, washed with phosphate buffered saline (PBS) and lyophilized prior to lipid analysis.

### Lipid analysis

Lipid analysis was performed at the Lipidomics Core of the Medical University of South Carolina using HPLC/MS-MS as previously described [13,14]. All values are reported normalized to mg protein as determined using the Lowry method [15].

### RT-PCR

Total RNA from lymphoblasts was prepared using TRI reagent (Sigma Aldrich) and equal amounts were reverse transcribed using ImProm-II (Promega, Wisconsin, USA) reverse transcriptase kit with a hexamer mixture as the template primer according to the manufacturer's instructions. Primer sequences for PCR analysis available upon request.

## Results and discussion

**Figure 1:**
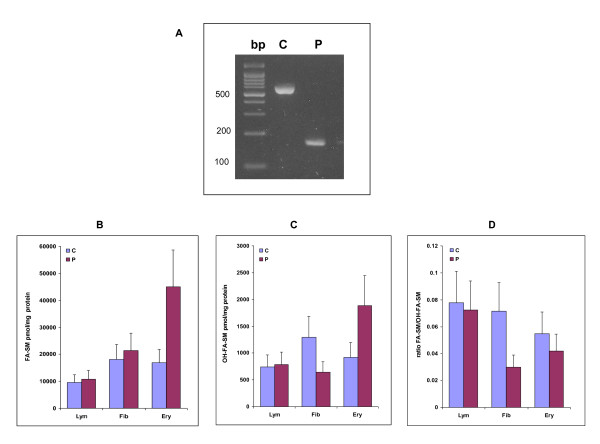
**Lymphocyte FA2H patient transcript and total FA-SM/OH-SM profiles**. A, Lane C shows the transcript obtained in control lymphocytes using the FA2H primer and lane P shows the shorter transcript obtained in patient lymphocytes. B, Total level of FA-SM in control and patient fibroblasts, lymphocytes and erythrocytes was quantitated by HPLC/MS-MS. C, Total level of OHFA-SM in control and patient fibroblasts, lymphocytes and erythrocytes was quantitated by HPLC/MS-MS. D, The ratio of the total OHFA-SM content divided by the total FA-SM content was calculated (mean of 2 experiments +/- SD).

We have previously shown that the c.786+1G→A mutation causes mis-splicing leading to skipping of exons 5 and 6 in fibroblasts. RT-PCR was perfomed in order to verify the exon skipping in lymphocytes. The expected 426bp shorter transcript, obtained in the patient, corroborated the exon skipping as shown in Figure 1A.

Subsequently we proceeded to investigate the sphingomyelin (SM) profiles in these cells as well as in erythrocyte membranes and fibroblasts (Figure 1B-C).

The total SM-fatty acid content in patient fibroblasts and lymphocytes was not significantly different from that of control subjects, while patient erythrocytes had a relatively higher SM-fatty acid content (Figure 1B). SM-hydroxy fatty acid content in patient lymphocytes was not significantly different from that of the controls, however it was reduced by 50% in patient fibroblasts and increased in patient erythrocytes (Figure 1 C). The decrease in SM-hydroxy fatty acid content in fibroblasts was especially evident when the ratio of SM-hydroxy fatty acid to SM-fatty acid was calculated. In patient erythrocytes this ratio was also significantly decreased as SM-hydroxy fatty acids were decreased relative to SM-fatty acids Figure 1D).

**Figure 2:**
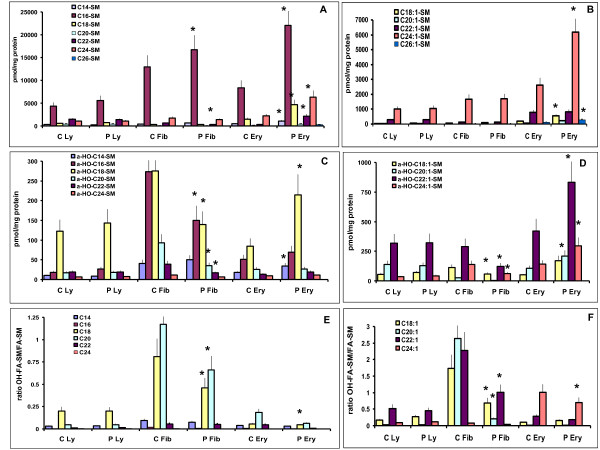
**Levels of saturated and unsaturated FA-SM and OHFA-SM in the range C14-C24 in fibroblasts, lymphocytes and erythrocytes of control subjects and in patients were quantitated by HPLC/MS-MS**. A, Saturated FA-SM. B, Unsaturated FA- SM.C, Saturated OHFA-SM. D, Unsaturated OHFA-SM. E, Ratio saturated OHFA -SM/FA-SM. F. Ratio unsaturated OHFA -SM/FA- SM (mean of 2 experiments +/- SD).

The distribution of the SM-fatty acid (Figure 2A, B) and SM-hydroxy fatty acids (Figures 2C, 2D) by chain length was investigated. C16 is by far the most abundant fatty acid in all cell types. No differences were seen between patient and control lymphocytes. SM-C16 fatty acid content was slightly increased in patient fibroblasts, and the levels of most SM-fatty acids were increased in patient erythrocytes. In all cell types, in control as well as patient, the distribution of SM-hydroxy fatty acids does not mirror that of their fatty acid counterpart. In lymphocytes, SM-OHC18 is the most abundant species, and the distribution of SM-hydroxy fatty acids is the same in the patient and the control. However, patient fibroblasts contain approximately 50% of SM-OHC16 and SM-OHC18 of the control values, and all the other SM-hydroxy fatty acid levels were significantly lower. The patient erythrocytes had an elevated content of all SM-hydroxy fatty acids, but with a significant increase of SM-OHC18 vs SM-OHC16.

The fraction of hydroxylated fatty acid relative to fatty acid (OHFA-SM/FA-SM) of the same chain length was calculated (Figure 2E, 2F). This ratio is the same for both control and patient lymphocytes, where the ratio is highest for C18. In contrast, in patient fibroblasts, the fraction of hydroxylated C20:1 is only 1/10 that of the control value, with reductions for C18 and C20 as well. C20 is the most hydroxylated saturated fatty acid in both control and patient, whereas C20:1 is the most hydroxylated monounsaturated acid in the controls and C24:1 the most hydroxylated monounsaturated acid in the patient. In erthrocytes, the ratio is decreased for the patient vs the control for all chain lengths, most noticeably for C20 and C22, with C20 and C24:1 the most heavily hydroxylated acids.

As SM is the major sphingolipid in human cultured fibroblasts [16], and taking into account the limited amount of sample, we focused our studies on this sphingolipid in cells obtained from patients using minimally invasive sampling. The lack of difference in the SM fatty acid and hydroxy fatty acid profile between patient and control lymphocytes was unexpected. Apparently the lack of FA2H as demonstrated by RT-PCR had no effect. On the other hand, SM hydroxy fatty acid content in fibroblasts was clearly decreased, with respect to both the total content and the fraction of hydroxylated SM. The high OHFA content but low OHFA-SM/FA-SM ratio in erythrocytes suggests an imbalance, rather than a quantitative change. The distribution of OHFA according to chain length is different in the patient and control fibroblasts, with C20:1 being the most hydroxylated acid in the controls and C24:1 the most hydroxylated in the patient.

The results we obtained for FA content and distribution confirm previous results [16] however in that paper the authors did not find any hydroxy fatty acids. They conclude that if present, they must be in amounts below their detection limit of a few pmoles. This is certainly not in agreement with the results presented here. The discrepancy could be due to differences in the sample preparation as well as analytical conditions.

The characterization of the FA and OHFA profiles was carried out also to identify differences between controls and patients which could possibly form the basis for a biochemical diagnostic test. The most significant difference was found in the relative decrease in OHFA-SM relative to FA-SM content in patient erythrocytes vs control. In cases where a skin biopsy is performed and fibroblasts are available, the decrease in saturated OHFA content coupled with the favoring of OHC24:1 in patients vs OHC20:1 in controls could also serve as a marker. Although genetic analysis is more feasible, FA-SM analysis could complement the molecular data and provide valuable information where genetic analysis is uninformative.

It is somewhat surprising that patient cells still contain a measurable amount of hydroxy fatty acids at all, considering the deleterious nature of the mutation in the FA2H gene. This would suggest presence of other enzyme/s with overlapping substrate specificity with FA2H. In fibroblasts and erythrocytes, which show a difference between the patient and the control, the 2-hydroxylating activity may be due to FA2H and to an additional enzyme with a different chain length preference. In lymphocytes, FA2H may be absent altogether while the proposed other enzyme performs the FA2-hydroxylation. Notably we were unable to detect any FA2H enzymatic activity in normal lymphocytes (results not shown).

## Conclusions

We conclude that OHFA-SM is decreased in patient fibroblasts and erythrocytes. Differences in FA-SM and OH-SM between patients and controls could serve as the basis for a diagnostic test.

## List of abbreviations

FA2H: Fatty acid 2-hydroxylase; FA: Sphingomyelin:SM, Fatty acid; OHFA: Hydroxy fatty acids; GalC: galactosylceramide.

## Competing interests

The authors declare that they have no competing interests.

## Authors' contributions

AR and PD prepared the samples, interpreted the results and prepared the manuscript, SE cared for the patients, HH and JB were responsible for the lipid analysis. All authors have read and approved the final manuscript.
